# Can Leader–Member Exchange Contribute to Safety Performance in An Italian Warehouse?

**DOI:** 10.3389/fpsyg.2017.00729

**Published:** 2017-05-11

**Authors:** Marco G. Mariani, Matteo Curcuruto, Mirna Matic, Paolo Sciacovelli, Stefano Toderi

**Affiliations:** ^1^Department of Psychology, University of BolognaBologna, Italy; ^2^School of Social Sciences, Leeds Beckett University, City CampusLeeds, UK

**Keywords:** safety climate, leader–member exchange, safety performance, safety participation, warehouse

## Abstract

**Introduction:** The research considers safety climate in a warehouse and wants to analyze the Leader–Member Exchange (LMX) role in respect to safety performance. Griffin and Neal’s safety model was adopted and Leader-Member Exchange was inserted as moderator in the relationships between safety climate and proximal antecedents (motivation and knowledge) of safety performance constructs (compliance and participation).

**Materials and Methods:** Survey data were collected from a sample of 133 full-time employees in an Italian warehouse. The statistical framework of Hayes (2013) was adopted for moderated mediation analysis.

**Results:** Proximal antecedents partially mediated the relationship between Safety climate and safety participation, but not safety compliance. Moreover, the results from the moderation analysis showed that the Leader–Member Exchange moderated the influence of safety climate on proximal antecedents and the mediation exist only at the higher level of LMX.

**Conclusion:** The study shows that the different aspects of leadership processes interact in explaining individual proficiency in safety practices.

**Practical Implications:** Organizations as warehouses should improve the quality of the relationship between a leader and a subordinate based upon the dimensions of respect, trust, and obligation for high level of safety performance.

## Introduction

In 28EU countries, transportation and storage sector got the third rate of Fatal and non-fatal accidents at work (Eurostat, 2015). In US, Occupational Safety and Health Administration [OSHA] (2004) reported that the fatal injury rate for the warehousing industry is higher than the national average for all industries. Warehouse is a workplace that can be hazardous in numerous ways, such us: uunsafe use of forklifts; improper stacking of products; failure to use proper personal protective equipment; failure to follow proper lockout/tagout procedures; inadequate fire safety provisions; or repetitive motion injuries. There are rules and procedures that have to be respected for a safe job. In fact, workers have to collaborate and help colleagues to prevent injuries and supervisors have to monitor, instruct and inform subordinates to improve their own safety performance.

A wide range of experts in safety with different educational and professional backgrounds developed theories and practices that aim to reduce the number of injuries and accidents. One of the most widespread framework on this topic is the Griffin and Neal (2000) model that stands in a conceptual analysis of safety performance as individual work behavior for which proximal antecedents are safety motivation and knowledge of safety issues and distal antecedents regard the safety climate. Griffin and Neal (2000) original model doesn’t consider the quality of relationship between supervisors and subordinates in safety perspectives, and this could be important in a workplace where the activities of instruction and information are basic for safe behaviors. The Leader–Member Exchange theory (LMX), coined from social exchange theory, contemplates this relationship and analyses its impacts on attitudes and behaviors appraising aspects as respect, trust, and obligation (Graen and Uhl-Bien, 1995). The success of LMX theory stands in having demonstrated influences on various outcomes such as task performance, organizational commitment, employee’s satisfaction and turnover intentions (Zacher and Frese, 2011). In addition to this, research in high-risk environments suggests that high quality LMX relationships are associated with increased safety communication, increased subordinate safety commitment, and fewer accidents (Hofmann and Morgeson, 1999). Hofmann et al. (2003), with a more specific focus, found that LMX and safety climate are interrelated to define behaviors as safety citizenship.

Summing it up, the present research wants to integrate Hofmann et al. (2003) suggestions into the Griffin and Neal (2000) model on safety performance and study the role of LMX in a workplace, as a warehouse, where quality of relationships are basic for safety. Indeed, our study tries to improve Hofmann et al. (2003), model including safety motivation and knowledge of safety as mediators.

## Theoretical Framework

### Griffin and Neal’s Model on Safety Performance

Griffin and Neal (2000) presented a study providing a theoretical pathway for individual safety performance and distinguished among the components of safety performance and its proximal (motivation and knowledge) and distal (safety climate) antecedents.

With regard to the components of safety performance, based on theories of individual performance (e.g., Campbell et al., 1993), they showed the distinction between safety compliance and safety participation. The first is about adhering to safe work practices and, in a broader sense, indicates behaviors directly related to work tasks. Safety compliance has been defined as engaging in activities that are part of the formal work procedures (i.e., correct use of equipment) and “applying appropriate work practices to reduce exposure to potential hazards and injury” (Fugas et al., 2011, p. 68). When safety participation was spotlighted, it has become evident that these kind of behaviors can enhance safety within the work environment and, generally toward “the maintenance of overall safety system” (Griffin and Neal, 2000, p. 356); this class of behavior can predicted micro-accidents, property damage, near-miss events and lost-time injuries (Curcuruto et al., 2015; Saracino et al., 2015).

While those addressed before were some components of safety performance, also safety antecedents are elements of the safety conduct pathway; in this context, the most relevant are safety knowledge and safety motivation (Curcuruto et al., 2016). In their study, Griffin and Neal (2000) showed that both knowledge and motivation mediated the impact of employees’ perceptions of safety on individual safety behavior; the model has been recently validated across different national contexts (Barbaranelli et al., 2015).

In this perspective, safety is a process rather than a desired outcome and is only partially due to the experience of the worker. A modern idea of safety stands in an ongoing, multidimensional and multilevel effort from employees. In this context, a motivated worker is willing to put an extra effort for safety and attributes a special value to safety behaviors (Neal and Griffin, 2006). On the other hand, safety knowledge is presented as the extent to which employees have a clear idea of safety processes and correct procedures and behaviors (Braunger et al., 2013). Griffin and Neal (2000) considered safety knowledge and motivation (Mariani et al., 2015) determinants of safety performance and mediators between safety climate and safety performance too.

Safety climate is considered in Griffin and Neal model a distal determinant of safety performance based on theories of psychological climate in organizations (e.g., James and McIntyre, 1996).

According to Zohar, safety climate can be seen as employee perception of the priority an organization (or direct supervisor) placed on safety (Zohar and Luria, 2005; Griffin and Curcuruto, 2016). It is constituted by a unified set of cognitions (held by workers) regarding the safety aspects of their organization. These cognitions, according to Zohar (2002), were related to employee perception of the relative importance management places on safety.

While there was substantial concurrence on the definition of safety climate, the dimensionality of the construct is debatable. Some researchers argued for a mono-dimensional construct as expression of managerial commitment for safety (e.g., Clarke and Ward, 2006), on the other hand, others considered safety climate as multi-dimensional (e.g., Neal et al., 2000; Zohar and Luria, 2005).

Zohar and Luria’s (2005) study focused on first-level supervision, considering a three-factor structure consisting of Active Practices (Monitoring–Controlling), Proactive Practices (Instructing–Guiding), and Declarative Practices (Declaring–Informing). Johnson (2007) confirmed this tripartite safety climate model.

In a warehouse, caring practices are about supporting workers, compliance stands for underlying respect of rules; coaching behaviors include the development of competences and knowledge. In the present study, these dimensions were picked to measure supervisory behaviors, as a general dimension.

Therefore, the first main objective of the present research is to verify the model Griffin and Neal (2000) in a warehouse workplace.

**FIGURE 1:**
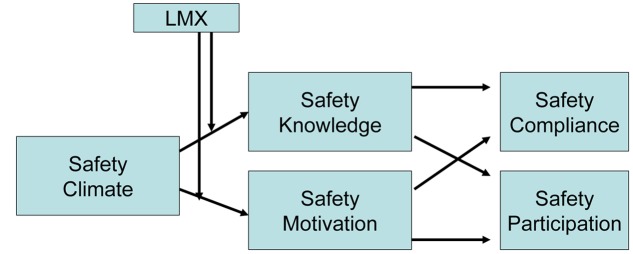
**The model of research**.

So, the hypotheses examine the mediations of motivation and knowledge (as proximal antecedents) between measures of safe and safety compliance and participation (**Figure 1**), following the Griffin and Neal (2000) model above presented.

H1a: *Safety knowledge has a mediational role in the relationship between safety climate and both safety compliance (i) and participation (ii).*
H1b: *Safety motivation has a mediational role in the relationship between safety climate and both safety compliance (i) and participation (ii).*

The hypotheses assign key roles to safety knowledge and safety motivation, as individual determinants for performances of safe behaviors; whereas safety climate has been adopted as organizational antecedent of the safety performance. The model therefore adopts, in a combined way, individual and organizational perspectives to explain safety performance.

### The Role of LMX in Safety

The positive role of leadership in safety was considered over the last two decades (e.g., Griffin and Hu, 2013). Leaders are relevant actors in safety as they can establish practices, rules and procedures – i.e, define organizational aspects of safety -, and communicate, motivate employees and give them good feedback for their work. In other words, they are expected to have an impact on safety antecedents and safety components. However, they are often called into question when research focuses on practices aimed to ensure safety on an organizational and formal level, while research on how they strive to achieve it on an informal level is more lacking – and cannot solely be about feedback. More precisely, there is one aspect on which safety research lacks most: the relationship the supervisor and the employee. This is the field of the LMX theory, coined from social exchange theory, which asserts that the unique relationship and exchange between the leader and the follower do impact on attitudes and behaviors of both (Scandura, 1999). LMX is a leadership framework that assesses the quality of the relationship between a leader and a subordinate based upon the dimensions of respect, trust, and obligation (Graen and Uhl-Bien, 1995) and its basis come from social exchange theory that claims how in a high-quality exchange, parties provide valuable assets to each other (Blau, 1964). Graen and Uhl-Bien (1995) explained this relationship in a precise way: “An offer will not be made and accepted without (1) mutual respect for the capabilities of the other, (2) the anticipation of deepening reciprocal trust with other and (3) the expectation that interacting obligation will grow over time as career-oriented social exchange blossom into a partnership” (p. 237). In fact, development of LMX is not based on a personal or friendship relationship; moreover, it is based upon the characteristics of the working relationship based upon the before mentioned dimensions (Graen and Uhl-Bien, 1995). High satisfaction of the follower from this relationship will have many positive impacts such as the overall satisfaction of the follower with the leader, increased follower performance, and follower positive organizational citizenship behavior (Zacher and Frese, 2011). On the other hand, if there is a low-quality LMX in which the leader only provides to the follower basic information that are necessary for performance and fulfilment of the job the follower performance and organizational citizenship will consequently be lower (Zacher and Frese, 2011). The basic idea behind LMX is that the leader develops two groups, the in-group and the out-group in an organizational context. To the in-group members the leader gives greater responsibility, more rewards and attention and has an elevated communication and a relationship based on trust, respect and mutual sense of obligation. In this case, the “in-group” members experience a higher quality leader–follower relationship and therefore they are prone to see the leader in a more positive way concluding how the leader is making an investment in them (Pierce et al., 2003). They feel like this high-quality relationship is based on the “rules of reciprocity” and they develop an “obligation” to give something back to the leader. Basically, they feel as if the leader invests in them, they should engage in actions and behavior that the leader values. In according to this, we believe that a high-quality relationship will be related to positive behaviors concerning safety performance. In fact, LMX has an impact on subordinates in a way that they will expend their roles beyond what is formally expected by engaging in citizenship behaviors oriented around safety and focus on improving safety performance of other team members and the organization (Hofmann et al., 2003).

In the present study, we want to provide a contribution on how the quality of the relationship between leaders and organizational members influences safe behaviors, as well as evidences on LMX were sought. This investigation was held to provide a theoretical model for our research that is collecting empirical evidences for our questions and hypotheses. Precisely, basing on the model provided by Griffin and Neal (2000), the objective is to provide evidence for the moderating role of LMX on the relationship between safety climate and safety compliance and participation. The *ratio* is that safety climate is related to the employee perception of the relative importance that management places on safety. Obviously, if we want that safety values and suggestions of supervisors are collected by employees it is necessary that the relationships between leader and members have a good quality. **Figure 1** shows the model of the research that follows the results of the Griffin and Neal (2000) research and the suggestions of Scandura (1999), presented above and forms the following hypothesizes:

H2a: *The LMX has a moderating role on the relationship between Safety climate and Safety knowledge. This last relationship exists where there is a high LMX.*
H2b: *The LMX has a moderating role on the relationship between Safety climate and Safety motivation. This last relationship exists where there is a high LMX.*
H3a: *The LMX has a moderating role on the mediational role of safety knowledge on safety compliance (i) and participation (ii) (moderated mediation). The relationships exist where there is a high LMX.*
H3b: *The LMX has a moderating role on the mediational role of safety motivation on safety compliance (i) and participation (ii) (moderated mediation). The relationships exist where there is a high LMX.*

The second set of hypotheses regards the role of working relationship between the supervisor and the employee. Our research hypothesis explores how the quality of the LMX can change the strength of climate effects on safety knowledge/motivation as well as the strength of indirect effects of safety climate on performing safe behaviors.

## Methodology

### The Context of the Research and Participants

Our research was set in a warehouse of great-size engineering company based in north Italy, which produces a great variety of industrial objects: from small fasteners to wheels for agricultural machines. The warehouse was structured in two basic functions, inbounding (materials that is received) and out bounding (materials that is sent outside). The roles were dived based upon the working location as following: planography/putaway 16%; picking 15%; pedestrian area 11%; loading 8%; working in more than one zone 8%; packing 7%; receiving 5%; internal packaging 3%, quality 3%; maintenance 3%; other 10%; no answer 13%. Hiendrich’s pyramid for 3 year (2011–2013) showed the following data: 66 first aids, 6 lost time accidents and zero sever, and fatal injuries. Safety is conceived in function of a prescribed set of behaviors: workers are explicitly advised about norm and sanctions. A particular example of this safety management is offered by the surveillance patrols that supervise the workplace in order to prevent violations.

The sample size consisted of 133 workers (74% of the warehouse population); 53% worked from zero to fifteen years in the organization and 14% had a role in safety practices (e.g., emergency team).

#### Procedure

A structured anonymous questionnaire was used to collect the data. Trained research assistant psychologist in a pencil-and paper format administered the questionnaire.

The study assured to respondents anonymity and confidentiality. The questionnaire included a statement regarding the personal data treatment, in accordance with the Italian privacy law (Law Decree DL-196/2003). The workers authorized and approved the use of anonymous/collective data for possible future scientific publications.

Because the data was collected anonymously and the research investigated psychosocial variables not adopting a medical perspective, ethical approval was not sought.

In the introduction part of the study it was explained how there is no right or wrong answer and how the answer should be as honest as possible. The participants completed the questionnaires privately and voluntarily in the workplace. The age and gender were not asked in order to guarantee the anonymity of the participants.

### Measures

The ZSCQ (Zohar and Luria, 2005) version of 11 items, validated by Johnson (2007), was used to measure safety climate through the employee perception of the priority that manager places on safety. Respondents expressed an opinion on a five-point Likert scale representing levels of agreement. For construct validity, since that the number of participants didn’t permit the adoption of confirmatory factor analysis with an 11-item scale (Bentler and Chou, 1987), the only exploratory factor analysis, with principal axis factoring extraction method, was performed. Results shows a one-factor structure that explains the 53.5% of variance and an alpha coefficient of.91.

The LMX-7 scale for Subordinate (LMX, Scandura and Graen, 1984) was adopted to evaluate the relationship of subordinates with his/her supervisor (dyadic exchange). The scale consists of seven questions, with a five-point Likert scale with a range of responses from “not a bit”, “a little”, “a fair amount”, “quite a bit”, “a great deal.” Some of the questions were: “How well does supervisor leader understand your job problems and needs?”. The exploratory factor analysis, with principal axis factoring extraction method, showed a one-factor structure that explained the 57.4% of variance. Moreover, the confirmatory factor analysis verified the one-factor model (GFI = 0.90, AGFI = 0.95, CFI = 0.97, RMSEA = 0.08). Previous researches, with Italian samples, showed an excellent Cronbach’s alpha (i.e., Portoghese et al., 2012). In this research alpha of Cronbach was 0.87.

Toderi et al. (2015) Italian version of Griffin and Neal model scales was adopted to measure Motivation, Knowledge and the components of the safety performance, that are compliance and participation. Every measure consisted in four items that used a five-point Likert scale to record the participant’s opinion. Cronbach’s alpha was 0.90 for Knowledge, 0.89 for motivation, 0.87 for compliance and 0.72 for participation.

The adaptation of the *LMX-7 scale for Subordinate* and of ZSCQ, version of Johnson (2007), to Italian language was done taking into account the standards recommended by the International Test Conference when adapting an instrument to a foreign language (Hambleton and Zenisky, 2011). The scales was firstly translated into Italian by two translators who were fluent in Italian and English. The translations were discussed with five experts, and some corrections were made. The back translation was conducted by two bilingual professors with no previous knowledge of the scale. This back-translated version was compared with the original English version.

However, a qualitative pilot study was carried out with six employees from the companies to evaluate the language forms and ensure a proper understanding of the all scales.

The questionnaire added some socio-organizational variables as the working sector and the years that an employee spent in the organization. In addition to this, focus groups with six members from the company were organized in order to check the questionnaire translation and to investigate the clarity and the pertinence of the items respect to workplace. Appendix 1 shows the items of scales which were used in the present research.

### Data Analysis

The statistical analysis plan consisted of the following steps: (1) calculation of the descriptive statistics, explorative factor analysis, confirmatory factor analysis, alpha of Cronbach and correlation indexes of the variables; (2) examination of the common method effect; (3) test of hypothesis 1a and 1b by mediation analysis; (4) test of hypothesis 2a and 2b by moderator analysis; (5) test of hypothesis 3a and 3b by moderated mediation analysis.

The degree to which common-method variance could be a threat to our analyses was analyzed, because a one-wave self-report design was used. Harman’s single-factor test by Confirmative Factor Analysis (CFA) was performed on six variables to verify the hypothesis that a single factor can account for all of the variance in our data (Podsakoff et al., 2003). Asymptotically distribution-free method, as implemented in AMOS software, was adopted because the variables could have not-normal distribution (**Table 1**) and CFI and RMSEA indexes were used to analyze the model fit.

**Table 1:** Correlations (Pearson’s *r*) and descriptive statistics of the scales (*N* = 133).

	1	2	3	4	5	6
*1. Safety climate*	(0.90)					
*2. LMX*	0.65^∗∗^	(0.87)				
*3. Safety Knowledge*	0.18^∗^	0.23^∗∗^	(0.90)			
*4. Safety Motivation*	0.08	0.06	0.47^∗∗^	(0.89)		
*5. Performance Compliance*	0.13^∗^	0.03	0.56^∗∗^	0.61^∗∗^	(0.87)	
*6. Performance Participation*	0.34^∗∗^	0.28^∗∗^	0.28^∗∗^	0.41^∗∗^	0.39^∗∗^	(0.72)
Mean	3.56	3.72	4.29	4.05	3.20	3.56
Standard deviation	0.98	0.80	0.69	0.75	0.81	0.98
N. of items	11	7	4	4	4	4

*Cronbach’s alpha in brackets; ^∗^*p* < 0.05, ^∗∗^*p* < 0.01, ^∗∗∗^*p* < 0.001*.

PROCESS (2.13 vers,), a macro for SPSS developed by Hayes (2013), was adopted to test simple meditation (Hypothesis 1), simple moderation (Hypothesis 2), and moderated mediation (Hypothesis 3). The variables in the proposed model were mean centered to minimize multi-collinearity. Simple Mediation (model n.4 of PROCESS macro), simple Moderation (model n.1 of PROCESS macro) and moderated mediation (model n.7 of PROCESS macro) were tested using the contemporary bootstrapping technique described by Hayes (2013), 5000 resampling with replacement. Bootstrapping was adopted because it provides not only a more reliable estimate of indirect effects; moreover, it does not make the often-unrealistic assumption about normality in the sampling distribution (Hayes, 2013). Additionally, this method is appropriate when sample sizes are relatively small (Hayes, 2013) because it produces a distribution using the observed data, from which statistical effects are estimated. This method was considered more reliable than a non-bootstrapping approach in the current sample, owing to the fact that 133 cases were included in the analysis.

In addition to this, bootstrapping method also has higher power and better Type I error control compared to other mediation analyses (Preacher and Hayes, 2008). Significance was determined by examining the 95% confidence interval produced by bootstrapping mediation analyses. In order for the mediation model to attain significance, the confidence interval must not include zero.

The total number mediation models, as the total number of moderated mediation models stressed throughout this research, were four: we had one independent variable (Safety climate), two mediators (safety motivation and knowledge) and two dimensions for safety performance, acting as dependent variables (safety compliance and participation). In moderated mediation model there was one moderator (LMX) too.

## Results

### Preliminary Analysis

**Table 1** shows descriptive statistics, alpha of Cronbach and correlation indexes for research variables. Results show that some skewness and kurtosis indexes have values higher than two standard errors, highlighting a significant difference respect to normal distribution (**Table 1**); substantially good is the internal homogeneity of the scales, as measured by alpha of Cronbach, and all positive are the relationships among the six variables.

Before testing our hypothesis, and considering the one-wave self-report design of the study, the common-method variance bias was analyzed. Harman’s single-factor test on common-method variance showed fit indexes not adequate for only one factor model (CFI = 0.74; RMSEA = 0.16). So, the hypothesis that common method variance could explain a substantial amount of covariance among variables was rejected. Therefore, variance is attributable to the constructs of the measures rather than to the measurement method.

### Mediation and Moderation Analysis

**Table 2** shows the main results of four mediation analyses. Bootstrap analysis showed that the indirect effects of Safety climate, via Knowledge, on Performance compliance and on Performance participation were significantly different from zero. Direct effect of Safety climate on Performance participation was significantly different from zero too. Moreover, results showed that the indirect effects of Safety climate, via Motivation, on Performance compliance and on Performance participation were not significantly different from zero. The direct effect of Safety climate on Performance participation was significantly different from zero in this case as well. These results confirmed H1 but hot H1b.

**Table 2:** Mediation analysis (*N* = 133).

		Direct effect					Indirect effect			
Mediator	Dependent variable	Effect	SE	T	LLCI	ULCI	Effect	Boot SE	Boot LLCI	Boot ULCI
*Safety Knowledge*	*Performance Compliance*	0.023	0.059	0.388	-0.094	0.139	0.076	0.041	0.004	0.165
*Safety Knowledge*	*Performance Participation*	0.269	0.067	4.012^∗∗∗^	0.136	0.4014	0.032	0.028	0.001	0.105
*Safety Motivation*	*Performance Compliance*	0.067	0.055	1.234	-0.041	0.176	0.031	0.040	-0.047	0.113
*Safety Motivation*	*Performance Participation*	0.282	0.065	4.365^∗∗∗^	0.154	0.409	0.019	0.023	-0.018	0.075

*^∗^*p* < 0.05, ^∗∗^*p* < 0.01, ^∗∗∗^*p* < 0.001*.

To test the H2a hypothesis that the Safety Knowledge problem is a function of Safety climate, moderated by LMX, a hierarchical multiple regression analysis was conducted.

**FIGURE 2:**
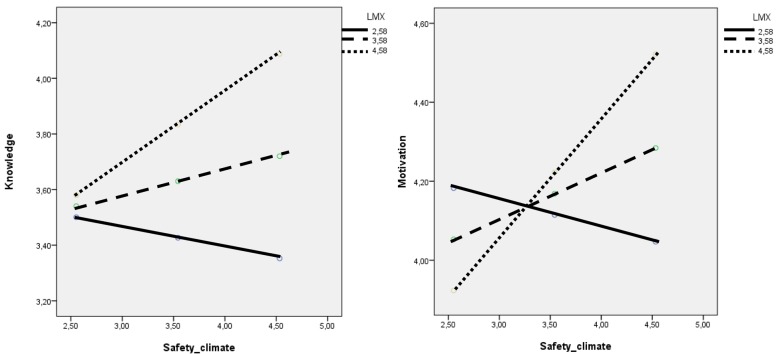
**Moderation analysis (*N* = 133)**.

The interaction term between Safety climate and LMX accounted a significant proportion of the variance in Knowledge, Δ*R*^2^ = 0.06, Δ*F*(1,132) = 7.36, *p* = 0.008, *b* = 0.17, *t*(132) = 2.71, *p* < 0.01. Examination of the interaction plot (**Figure 2**) showed an effect that increases when LMX increases: only at high levels of LMX, Safety climate influences Knowledge (Effect = 0.26; *SE* = 0.12; *t* = 2.06; *p* < 0.05; LLCI = 0.01; ULCI = 0.50).

The same procedure was adopted to test H2b hypothesis. The interaction term between Safety climate and LMX accounted a significant proportion of the variance in Motivation, Δ*R*^2^ = 0.09, Δ*F*(1,132) = 11.87, *p* = 0.001, *b* = 0.19, *t*(132) = 3.45, *p* < 0.001. Examination of the interaction plot (**Figure 2**) showed an effect of Safety climate on Motivation that increases when LMX increases: only at high levels of LMX, Safety climate influences Motivation (Effect = 0.30; *SE* = 0.11; *t* = 2.76; *p* < 0.01; LLCI = 0.09; ULCI = 0.52).

So, these evidences confirmed the H2a and H2b hypothesis.

### Moderated Mediation Analysis

Four moderated mediation analyses were performed to test hypotheses 3a and 3b, results are presented in **Table 3**.

**Table 3:** Moderated mediation analysis: conditional indirect effect(s) of X on Y (*N* = 133).

		LMX low		LMX medium		LMX high		Index of moderated mediation			
Mediator	Dependent variable	Boot LLCI	Boot ULCI	Boot LLCI	Boot ULCI	Boot LLCI	Boot ULCI	Index	SE (Boot)	Boot LLCI	Boot ULCI
*Safety Knowledge*	*Performance Compliance*	-0.145	0.067	-0.039	0.154	0.016	0.289	0.087	0.037	0.022	0.171
*Safety Knowledge*	*Performance Participation*	-0.083	0.025	-0.012	0.093	0.003	0.169	0.037	0.023	0.006	0.096
*Safety Motivation*	*Performance Compliance*	-0.156	0.063	-0.018	0.185	0.079	0.342	0.125	0.034	0.064	0.197
*Safety Motivation*	*Performance Participation*	-0.103	0.028	-0.004	0.113	0.039	0.212	0.064	0.025	0.025	0.125

Bootstrap analysis showed that Safety climate had an indirect effect on performance compliance and on performance participation through Knowledge with a high level of LMX. Whereas the product terms were significantly different from zero at high levels of LMX, them were not significantly different from zero at low and medium levels of LMX (**Table 3**). These evidences confirm hypothesis 3ai and hypothesis 3aii.

Similar results regard the second block of analysis. Safety climate had an indirect effect on performance compliance and on performance participation through Motivation with a high level of LMX. There were a significantly differences from zero of product terms at high levels of LMX, whereas them was not significantly different from zero at low and medium levels of LMX (**Table 3**). In fact, these evidences confirm hypothesis 3bi and hypothesis 3bii.

## Discussion

### Conclusion and Discussion

The present research aimed to apply a model of Griffin and Neal (2000) to investigate the impact of Safety climate on employee safety performance and to highlight the role of knowledge, motivation and LMX, in particular, on this path. In our study, indeed we wanted to observe the quality of the relationship between leaders and employees and its impact on their resources for safety performance. The first set of Hypotheses considered Safety knowledge and safety motivation as mediators in the relationship between safety climate and both safety compliance and participation.

We found that knowledge mediated the relationship between safety climate and safety performance (compliance and participation) following the model of Griffin and Neal (2000). This result supported Hypothesis 1a. On the other hand, motivation didn’t play a mediation role, therefore Hypothesis 1b was not verified. However, the correlation indexes show that the role of knowledge and motivation, in respect to safety performance proved to be important following the suggestions of Campbell et al. (1993).

The second set of Hypotheses regarded the moderator role of LMX between safety climate and both safety knowledge and motivation. Both, hypothesis 2a and 2b were supported by results: LMX played a moderator role between safety climate and knowledge/motivation variables. High levels of LMX permitted to safety climate of influencing knowledge and motivation.

The third set of Hypotheses considered the moderator role of LMX in a model where *safety knowledge and motivation are mediators between safety climate and safety compliance/participation behavuours.* Moderated mediation analysis confirmed all of that: knowledge and motivation were mediators only when LMX showed high levels. Therefore, hypothesis 3a and 3b were confirmed for both performance indicators, compliance and participation.

All this takes on a stronger meaning if we think that the model of Griffin and Neal was also verified with Italian workers while not operating in warehouses (Barbaranelli et al., 2015).

The results of the study have a number of implications for research. First, the study has demonstrated that LMX has an important role in the safety performance. From a theoretical prospective, the results of LMX are substantially in line with evidences in different fields as well. For example, Ozer (2008) found a relationship between LMX and job performance. Significant relationships were reported between LMX and Organizational Citizenship Behavior too (Ilies et al., 2007). This evidence provides stronger argumentation toward the benefits associated to high quality LMX. From a more general perspective, the LMX theory appears to be as a comprehensive theoretical tool as it allows focusing not only on required behaviors, but also on discretionary and side behaviors. Last, we would like to consider briefly the impact of the specific content of LMX on behaviors and performance. Previous studies showed that the reciprocation of subordinates is consistent with leaders’ values and needs. An exchange aimed at giving value to safety issues is likely to cause further engagement in safety behaviors and performance (Hofmann et al., 2003, in Ilies et al., 2007). The value of correlation index between LMX and safety climate shows that the two constructs are related but separate; moreover, the level of this relationship is in line with correlation indexes between LMX and different type of climate that are presented in literature, as service climate (Auh et al., 2016). Finally, the LMX emerges as important contribution to the model although the climate measure was based on the supervisor’s behaviors and the constructs were conceptual similar but separate, In fact, the correlation indexes between the two measures confirmed that.

### Limitations of the Study

The limitations of this study can be found in some research design issues. The number of participants played a constraining role in the research design, as the only one organizational case, limits the generalization of the results. Moreover, for the limited participants, we took a measure of overall Safety climate and not specific dimensions as caring, compliance and coaching. We therefore chose to focus on the first kind and general measure of Safety climate to support the hypotheses. Furthermore, safety climate research has consistently presented a conceptual focus on collective phenomena, including both a focus on group and organizational level analyses (Griffin and Curcuruto, 2016). However, the present research, as the original study of Griffin and Neal (2000) and others that used their approach (i.e., Braunger et al., 2013) adopted an individual level to consider safety climate. In fact, our evidences can be compared with their work and a more-broad literature, focused on social-exchange mechanisms, entailed by LMX, which are typically related on the individual perception of the employees’ personal relationships with the organization and supervisors. Furthermore, the present study could not adopt a group-level climate analysis because the number of workgroups/sectors was very low.

Secondly, we adopted a cross sectional design aimed at collecting data from different work areas at a due time because company didn’t permit it. However, we have verified that the effect of common method is contained. We think that is a limited problem because the correlation indexes of variables have a wide range and in addition to this there was a relationship not significantly different from zero. Third, we analyzed the quality of the relationship between leaders and members but we did not examine the style of leadership, as transformational and transactional types. Fourth, this study adopted the performance as alone outcome without considers aspects of wellbeing of employees as job satisfaction (i.e., Guglielmi et al., 2016) moreover the performance was self-evaluated by workers rather than observed externally without considering supervisors’ observations. Hayes (2013) shows that the statistical method that our research adopted has limitations that could be overcome by Structural Equation Model (SEM), but the research sample size doesn’t permit the use of SEM (Bentler and Chou, 1987).

Finally, among the point of weakness there is the fact that the adopted measures of LMX-7 and ZSCQ were not validated in previous study. However, the psychometric quality (factorial validity and reliability) was found good with the data of the present research.

### Practical Implications

The results of the study shed light on the importance of a positive relationship between managers and workers in industrial context for safety purposes. They asserted that when the LMX is high (i.e., positive and enriching), safety behaviors among employees (thus, their compliance and participation) are rooted by specific safety-oriented motivation and knowledge. Conversely, when LMX is not as strong as previously shown, neither safety motivation nor knowledge do effectively affect safety performance. Thus, the way managers relate with co-workers can enrich their supervisory performance.

About warehousing in particular, according to the American Occupation Safety and Health Administration (OSHA), safety leaders should follow safety checklists that help identify and tap potential hazards. These checklists do particularly insist on materials handling safety, forklift safety and communication. There are many different communication issues about warehousing safety worth to be described. One the other hand, research suggest that employees will reciprocate implied obligation formed by LMX by expanding roles and behaving in accordance with behavioral expectations in regard to group safety climate (Hofmann et al., 2003).

Finally, LMX appears to assume an interesting role in safety procedures and we hope that LMX will have new space in the scientific safety research. A considerable amount of authors have already carried agreed that safety is not just the outcome of a mere technical and procedural pathway, moreover it is a process that involves people, their relations and their influencing abilities and values. The results of the study support that supervisors should improve their non-technical skill (Burke et al., 2007) and competence (Morone et al., 2016.) in relationships management. Organizational actors need to be given more evidences for the relations between safety performance and LMX, OCB and climate among employees. An intervention, focusing on the promotion of self and relational management as a primary prevention perspective could improve individual resources such as emotional intelligence (Di Fabio and Saklofske, 2014), to increase the level of LMX and safety performance (Di Fabio, 2014; Di Fabio and Kenny, 2016),

## Ethics Statement

The questionnaire included a statement about personal data treatment, in accordance with the Italian privacy law (Law Decree DL-196/2003). The workers authorized and approved the use of anonymous/collective data for possible future scientific publications. The ethical approval was not sought, for two main reasons: First, the data for this research were collected anonymously and workers authorized and approved the use of anonymous/collective data. Second and most important, the research is based on the study of psychosocial variables that refer to the work environment and its perceptions by workers. For this reason, the study cannot be considered a medical research or an experiment on human subjects that need ethical approval following the Recommendations from WMA Declaration of Helsinki – Ethical Principles for Medical Research – Involving Human Subjects (World Medical Association [WMA], 2013).

## Author Contributions

MM, MC, and ST conceptualized the study and chose the theoretical framework. The first version of the introduction was written by PS and MM. ST and MM analyzed the data and wrote the methods and results. MM, MC, and ST wrote the discussion and practical implications. All the authors then revised and improved the manuscript several times.

## Conflict of Interest Statement

The authors declare that the research was conducted in the absence of any commercial or financial relationships that could be construed as a potential conflict of interest.
